# Investigation of Factors Influencing the Occurrence of 3C-Inclusions for the Thick Growth of on-Axis C-Face 4H-SiC Epitaxial Layers

**DOI:** 10.3390/ma13214818

**Published:** 2020-10-28

**Authors:** Keiko Masumoto, Kazutoshi Kojima, Hiroshi Yamaguchi

**Affiliations:** National Institute of Advanced Industrial Science and Technology, Central 2 1-1-1 Umezono, Tsukuba 305-8568, Japan; kazu-kojima@aist.go.jp (K.K.); hiroshi-yamaguchi@aist.go.jp (H.Y.)

**Keywords:** silicon carbide, epitaxial layer, on-axis, spiral growth, 3C-inclusions

## Abstract

In this study, we grew homoepitaxial layers on 3-inch on-axis carbon-face 4H-silicon carbide substrates and attempted to suppress the generation of 3C-inclusions. It was found that the 3C-inclusion density decreased with increasing time spent on reaching an objective flow rate for the precursors. It is suggested that 3C-SiC nucleation occurred on large terraces of the on-axis substrates, which existed before the substrates were covered with spiral hillocks. This nucleation was suppressed owing to the decrease in the degree of supersaturation at the initial growth stage. Moreover, we found that the 3C-inclusions were also generated owing to contamination in the form of graphite products. Furthermore, we succeeded in growing a thick on-axis 4H-SiC homoepitaxial layer on a 3-inch substrate and demonstrating its free-standing epitaxial layer with a thickness of 182 μm and a 3C-inclusion density of 2.0 cm^−2^.

## 1. Introduction

Currently, silicon (Si) power devices are widely used; however, further improvements in the consumption efficiency and applicability of power devices are required. Silicon carbide (SiC) power devices have low energy loss and can be used at higher temperatures. Furthermore, owing to their physical properties—including a wide bandgap and high thermal conductivity [1]—SiC device-systems can be miniaturized. There are many polytypes of SiC, such as 4H, 6H and 3C. 4H-SiC is primarily used for power devices because of its higher electron mobility and wider bandgap [2,3]. Consequently, 4H-SiC power devices—such as Schottky barrier diodes and metal-oxide-semiconductor field-effect transistors (MOSFETs) with blocking voltages below a few kilovolts—are commercially used in air-conditioning equipment, train components, etc.

4H-SiC insulated-gate bipolar transistors (IGBTs), with blocking voltages above 10 kV, are expected to gain widespread use because they can reduce the size and losses in electronic components used in smart grids and high-voltage DC transmission systems [4,5]. In particular, obtaining n-channel SiC IGBTs is important because the effective mobility of the n-channel SiC MOS structure is higher than that of its p-channel counterpart [6]. Furthermore, the carrier lifetime in a lightly-doped n-type SiC epitaxial layer is longer than that in its lightly-doped p-type counterpart [7,8], which leads to the low on-resistance of n-channel SiC IGBTs. To fabricate n-channel SiC IGBTs, a p^+^ collector is required. Heavily acceptor-doped and low-resistivity p-type substrates would be used as the p^+^ collector to ensure the simplest and lowest-cost fabrication process if such p-type substrates could be obtained. Research on heavily Al-doped p-type 4H-SiC bulk crystal growth faces some significant challenges, such as improving the growth rate and suppressing 6H-SiC polytype inclusions [9]. Since low-resistivity and high-quality p-type bulk growth is still difficult, the structure of the n-channel SiC IGBT—including the p^+^ collector and thick n^-^ drift layer—can be ensured through epitaxial growth on a standard n-type substrate that is removed by polishing after the epitaxial growth [5]. Thus, a free-standing epitaxial layer is necessary for producing n-channel IGBTs.

4H-SiC epitaxial growth has been achieved by using step-flow growth on 4° off-angle substrates to enhance polytype stability [2,10]; however, two principle problems exist in the process of off-angle epitaxial growth, the first of which is basal plane dislocations (BPDs). It has been reported that the generation of single Shockley stacking faults, attributed to BPDs propagating from the off-angle substrates to the epitaxial layers, results in the degradation of bipolar device performance in the forward-voltage direction [11]. The BPDs in epitaxial layers must be removed to improve the yield of SiC IGBTs. The second problem involves the difficulty in thickening the epitaxial layers. It has been reported that high-density 3C-inclusions are easily generated at the wafer edge of the step-flow upstream side because the stacking information of the substrate cannot be applied at the wafer edge of the step-flow upstream side owing to the absence of steps; the generation area of the high-density 3C-inclusions increased with increases in epitaxial layer thickness [12]. The generation of these high-density 3C-inclusions at the wafer edge of the step-flow upstream side also should be suppressed to improve the yield of SiC IGBTs.

Spiral growth on on-axis SiC substrates resolves these problems. Firstly, in the on-axis substrates, BPDs do not propagate to the epitaxial layers because the growth direction is perpendicular to the basal plane. Furthermore, it has been reported that the generation of single Shockley stacking faults is not observed for PiN diodes fabricated using on-axis SiC epitaxial wafers [13]. Secondly, the generation of high-density 3C-inclusions at the wafer edge of the step-flow upstream side can be suppressed by using spiral growth because this type of growth originates from threading screw dislocations (TSDs), which eternally supply steps even though the thickness of the epitaxial layers increases. On-axis SiC substrates have small off-angles, and spiral growth occurred when the off-angle was less than 0.05° in our previous report [14]. Another group of researchers reported that spiral growth became the dominant mechanism when the off-angle was less than 0.15°, and both step-flow growth and spiral growth simultaneously took place when the off-angle was 0.15–0.5° [15,16,17]. Thus, it is clear that spiral growth tends to occur at lower off-angles, but the reason for the boundary off-angle where the spiral growth occurs is unknown. We previously investigated the conditions surrounding spiral growth and found that this type of growth occurred when the off-angle was less than the tilt angles of spiral hillocks, because the growth rate of spiral growth became higher than that of step-flow growth [14]. Consequently, we have succeeded in growing on-axis 4H-SiC homoepitaxial layers on the 3-inch substrates using spiral growth [14]. The next step to obtain high-yield n-channel SiC-IGBTs is the growth of thick, high-quality on-axis 4H-SiC epitaxial layers using spiral growth.

The main problem when using on-axis 4H-SiC substrates is suppressing the 3C-inclusions, which will more likely be generated during on-axis epitaxial growth in comparison to 4° off-angle growth [14,16], although the presence of high-density 3C-inclusions attributed to the absence of steps at the wafer edge of the step-flow upstream side can be suppressed. It is known that two-dimensional nucleation tends to occur when large terraces exist [2], and the polytype of the nucleus is 3C-SiC, which is stable at chemical vapor deposition (CVD) growth temperatures of 1500–1700 °C [2,18]. On-axis 4H-SiC substrates have much larger terraces than 4° off-angle substrates, which leads to increased 3C-SiC nucleation. It is thought that the removal of generated 3C-SiC nucleation and the suppression of 3C-SiC nucleation itself are effective in mitigating the creation of 3C-inclusions. From this perspective, the addition of chlorine gas is a good method to remove generated 3C-SiC nucleation because it has been reported that higher chlorine content in gas mixtures preferentially etches 3C-inclusions [16]. To suppress 3C-SiC nucleation itself, the degree of supersaturation must be reduced. This means that increases in growth rate are difficult to establish because 3C-SiC nucleation occurs more easily at high growth rates, owing to higher degrees of supersaturation. It has been reported that on-axis silicon-face (Si-face) epitaxial growth rates are limited to below 10 μm/h to suppress the 3C-inclusions, irrespective of the addition of HCl, in both the spiral and step-flow growth modes [19]. However, the growth rate should be increased to grow thick epitaxial layers. In contrast, the growth rate of 2 × 2 cm^2^ on-axis Si-face epitaxial layers was increased to 100 μm/h by using a particular chlorinated precursor—methyltrichlorosilane (MTS)—but this high growth rate could be obtained only in the upstream area of the reaction chamber because of an abrupt depletion effect that was attributed to MTS [20]. It is thought that MTS is not suitable for large diameter growths. Therefore, we focused on on-axis 4H-SiC carbon-face (C-face) epitaxial growth for increasing the growth rate. It has been reported that step bunching seldom occurs in C-face epitaxial growth in comparison to the Si-face in step-flow growth because the surface energy of the C-face is smaller than that of the Si-face [2,21]. This means that the steps on the spiral hillocks of the C-face on-axis 4H-SiC epitaxial layer also have fewer occurrences of bunching in comparison to the Si-face. It has been reported that the step height near the top of the spiral hillocks at the C-face was 0.5 nm corresponding to a half-unit cell [14], and those at the Si-face was 1 nm corresponding to a one-unit cell [20]. Therefore, the terrace widths of the spiral hillocks on the C-face must be narrower than those of the Si-face during on-axis epitaxial growth. We expected that the on-axis C-face growth rate would increase concurrently with the suppression of 3C-inclusions.

We also focused on a process for fabricating a free-standing epitaxial layer, which is usually conducted by polishing substrates, as described above [5]. This process wastes the substrate material, increasing the cost of SiC-IGBTs. A SiC ingot slicing method, referred to as the KABRA process—where substrates are peeled off using a separation layer formed by an irradiating laser from the upper surface of the ingot—has been developed by the DISCO Corporation [22,23]. We applied this process to fabricating the free-standing epitaxial layer because the substrate that separates from the epitaxial layer can be reused.

In this study, we first attempted to suppress the generation of 3C-inclusions in the 4H-SiC C-face on-axis epitaxial layers by optimizing growth conditions; this was done to investigate the source of the 3C-inclusions. Next, the thick on-axis 4H-SiC epitaxial layer was grown, and the thick free-standing epitaxial layer was demonstrated by separating it from the substrate using the KABRA process.

## 2. Experimental Procedure

**Figure 1:**
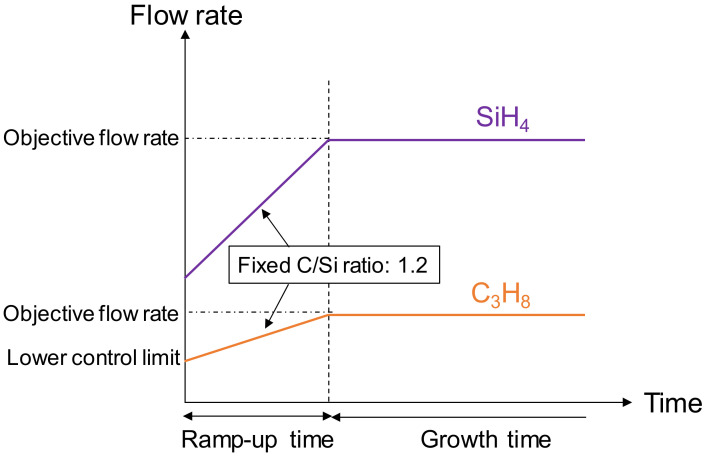
Growth process diagram of on-axis 4H-SiC epitaxial layers showing ramp-up time of the precursors flow rates.

Homoepitaxial layers were grown on commercially available 3-inch C-face 4H-SiC substrates (SICC and Sicrystal) with surface orientations close to perfectly on-axis (less than 0.05° off-angle). A vertically blown CVD system, where substrates were oriented perpendicular to the gas flow, was used for this purpose [24]. H_2_ (9N) was used as the carrier gas, SiH_4_ (5N) and C_3_H_8_ (5N) were used as the precursors, N_2_ was used as the doping gas and HCl (5N) was used as the additive gas. The epitaxial growth was carried out at 1580–1590 °C and 2.7 kPa. The growth rate was either approximately 20 or 40 μm/h. The H_2_ flow rate was 30 slm. The SiH_4_ and C_3_H_8_ flow rates were 27 and 10.6 sccm, respectively, at the growth rate of 20 μm/h, and 54.1 and 21.2 sccm, respectively, at the growth rate of 40 μm/h. The C/Si ratio was maintained at 1.2. The N_2_ flow rate was 2 sccm, and the HCl flow rate varied between 0 and 810 sccm. We also varied the time spent on reaching the objective flow rate of the precursors (ramp-up time) from 5 to 90 min. Figure 1 shows a growth-process diagram explaining the ramp-up time. The flow rate of C_3_H_8_ increased from the lower control limit of the mass flow controller to the objective value, and that of SiH_4_ was controlled to maintain a fixed C/Si ratio. The carrier concentration of the n-type epitaxial layer, obtained through capacitance–voltage measurements, was approximately 3–7 × 10^15^ cm^−3^.

Surface observation of the on-axis 4H-SiC epitaxial layers was carried out using Nomarski optical microscopy (NOM; OLYMPUS BX51, Tokyo, Japan) and tapping-mode atomic force microscopy (AFM; Veeco Nanoscope, Plainview, NY, USA). The 3C-inclusion density was determined through photoluminescence (PL) images obtained using 295–370 nm bandpass Hg lamp excitation, whose power density was 4500 mWcm^−2^, and detected using a 750 nm long-pass filter at room temperature (PHOTON Design SemiScope, Tokyo, Japan). We confirmed that the 3C-inclusions exhibited dark contrasts in these PL conditions, as described in Ref. [25] by PL spectroscopy with the same setting used in our previous report [14]. PL images of wafer size were made by merging images, which were obtained by using microscope objective lens with magnification of 5×. The origin of the 3C-inclusion was observed using cross-sectional scanning electron microscopy (SEM; Carl Zeiss Merlin, Oberkochen, Germany), transmission electron microscopy (TEM; HITACHI H9000 UHR, Tokyo, Japan) and energy-dispersive X-ray spectroscopy (EDX; JEOL JED-2300, Tokyo, Japan).

## 3. Results and Discussion

### 3.1. Effects of HCl Addition on 3C-Inclusions

**Figure 2:**
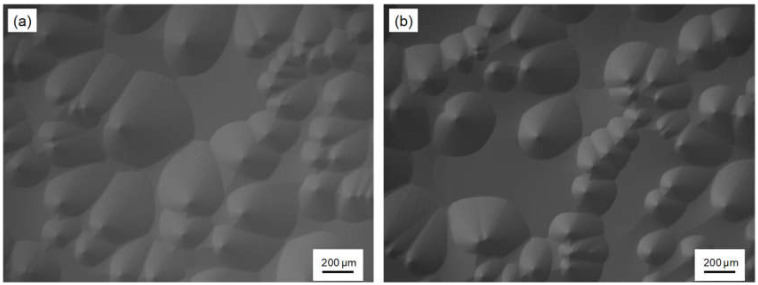
Nomarski optical microscopy (NOM) images of the on-axis 4H-SiC epitaxial layer surface grown (**a**) without and (**b**) with HCl addition. (**b**): The HCl flow rate was 540 sccm.

We first investigated the effect of HCl addition on the 3C-inclusion density because it has been reported that higher chlorine content in gas mixtures preferentially etches 3C-inclusions. The 3C-inclusion density gradually decreased with increases in the Cl/Si ratio, from 0 to 10, where a horizontal hot-wall CVD system was used [16]. We varied the HCl flow rate from 0 to 810 sccm at a SiH_4_ flow rate of 27 sccm; the Cl/Si ratio was varied from 0 to 30. The growth rate and time were 20 μm/h and 1 h, respectively. Figure 2a,b show NOM images of the on-axis 4H-SiC epitaxial layer surface grown without and with HCl addition, respectively. The HCl flow rate in Figure 2b was 540 sccm. These epitaxial layer surfaces are covered with spiral hillocks, and we confirmed that the epitaxial layers experienced spiral growth throughout the wafer, irrespective of the HCl flow rate.

**Figure 3:**
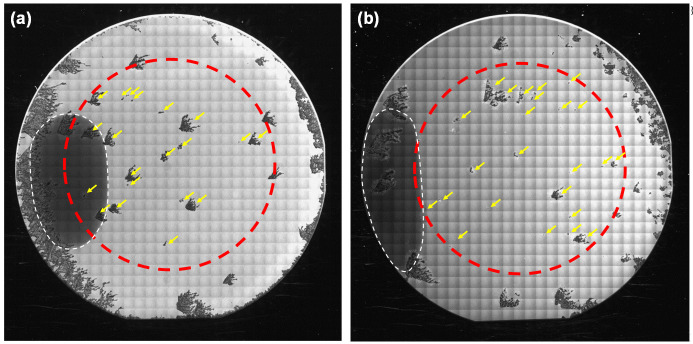
Photoluminescence (PL) images of the on-axis 4H-SiC homoepitaxial layers grown on the 3-inch substrates (**a**) without and (**b**) with HCl flow. (**b**) The HCl flow rate was 810 sccm.

**Figure 4:**
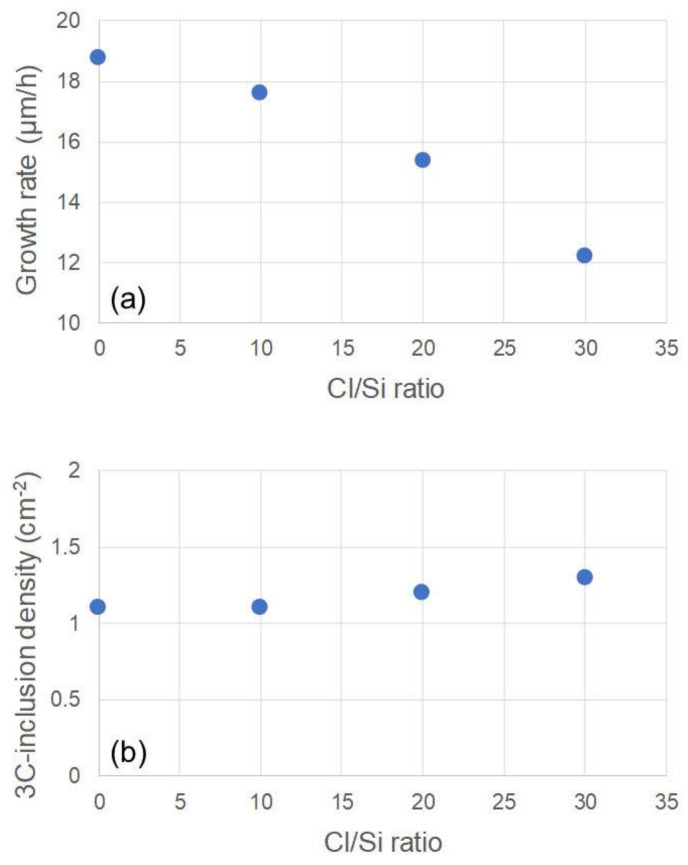
Dependence of (**a**) the growth rate and (**b**) the 3C-inclusion density of the on-axis 4H-SiC epitaxial layers on the Cl/Si ratio.

Figure 3a,b show PL images of the 4H-SiC homoepitaxial layers grown on the 3-inch substrates without and with HCl flow, respectively. The HCl flow rate in Figure 3b was 810 sccm. It is known that 3C-inclusions in 4H-SiC epitaxial layers exhibit dark contrasts in these PL conditions [25], as indicated by the yellow arrows. The dark ellipses encircled by white dashed lines are the facet areas of the substrates. The 3C-inclusion density was calculated in concentric 2-inch areas of the 3-inch wafers—shown in the red dashed circles—because high-density 3C-inclusions were occasionally generated from the wafer edge. The occasional high-density 3C-inclusions at the wafer edge were not due to the absence of steps at the wafer edge of the step-flow upstream side because they were generated at various positions of the wafer edge, as shown in Figure 3. Figure 4a,b show the dependence of the growth rate and the 3C-inclusion density, respectively, on the Cl/Si ratio. We found that the growth rate decreases with increase in the Cl/Si ratio, especially when the Cl/Si ratio is over 10; furthermore, the 3C-inclusion density is approximately 1 cm^−2^, irrespective of the HCl flow rate. This indicates that the preferential etching of 3C-SiC by chlorine, reported in Ref. [17], did not occur, and the addition of HCl had no effect on the 3C-inclusion density in this experiment. We predicted that the etching selectivity between 3C-SiC and 4H-SiC was reduced when the etching rate for 4H-SiC was high. Using this vertically blown CVD system, the etching rate for 4H-SiC epitaxial layers was approximately 5 μm/h at 1590 °C in a H_2_ atmosphere. The etching rate was much higher than that when using traditional horizontal hot-wall CVD systems. For example, it has been reported that the etching rate of a horizontal hot-wall CVD system is about 0.4 μm/h at 1660 °C in a H_2_ atmosphere [26]. Moreover, the growth rate decreases with the increase in the HCl flow rates, as shown in Figure 4a. This makes it clear that more effective etching phenomena took place during epitaxial growth when using this vertically blown CVD system, in comparison to horizontal hot-wall CVD systems. Owing to the high etching rate for 4H-SiC, no effect of preferential etching for 3C-SiC by HCl was observed. It should be noted that SiC deposition on the chamber walls was reduced by the addition of HCl, leading to lower maintenance frequency. To maintain the growth rate, we used a Cl/Si ratio below 10 for subsequent growth experiments.

### 3.2. Effects of Increasing the Ramp-Up Time on 3C-Inclusions

**Figure 5:**
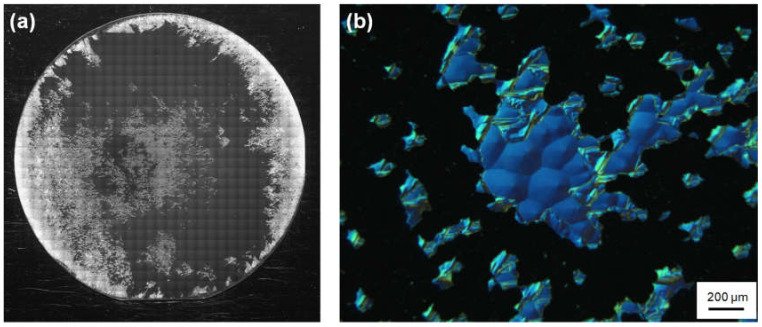
(**a**) PL image of the on-axis 4H-SiC homoepitaxial layer grown on the 3-inch substrate at the growth rate and ramp-up time of 40 μm/h and 5 min, respectively, and (**b**) NOM image at the central region.

It can be seen from the above results that it was difficult to suppress the 3C-inclusions using preferential etching for 3C-SiC in this experiment. Therefore, the suppression of 3C-SiC nucleation was necessary to decrease the 3C-inclusion density. Our previous research showed that spiral growth occurred when the off-angle was less than the tilt angles of the spiral hillocks [14]. This means that the terraces of the on-axis substrates were larger than those of the spiral hillocks. It is thought that 3C-SiC nucleation occurs more easily on terraces of the substrate surface in comparison to terraces of the spiral hillocks. To suppress 3C-SiC nucleation on the substrate surface, the degree of supersaturation before the substrate is covered with spiral hillocks should be decreased. To accomplish this, we increased the ramp-up time at the initial growth stage and investigated its effect on the 3C-inclusion density. We first grew an on-axis 4H-SiC epitaxial layer at a growth rate and ramp-up time of 40 μm/h and 5 min, respectively. The growth time was 30 min. The growth rate was double that of the experiment using HCl addition, described in the previous section, toward achieving the goal of thick growth. Figure 5a,b show a PL image of the 4H-SiC homoepitaxial layer on the 3-inch substrate and an NOM image at the central region, respectively. The 3C-inclusions are generated with a high density and with very rough morphology; in Figure 5a, they present a bright contrast owing to the scattering of the excitation light. Figure 5b shows that the epitaxial layer is composed of 4H-SiC spiral hillocks and high-density 3C-inclusions with black contrast. We found that the 3C-inclusion density increased with an increase in growth rate. It is thought that the degree of supersaturation at the initial growth stage was too high to suppress 3C-SiC nucleation on the on-axis substrates.

**Figure 6:**
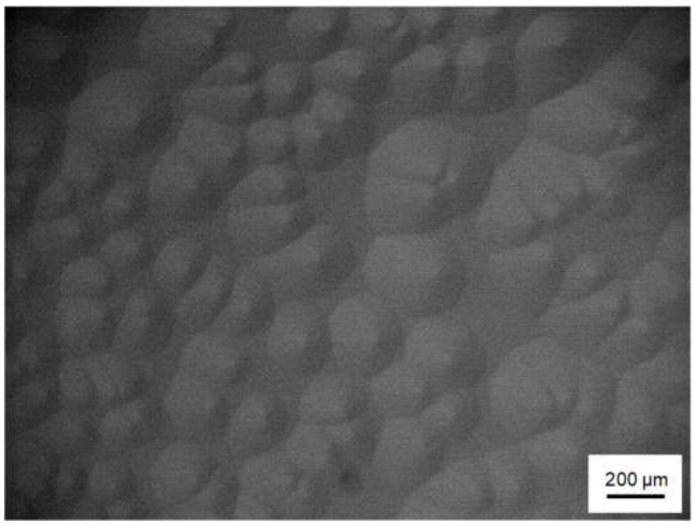
NOM images of the on-axis 4H-SiC epitaxial layer surface at the growth rate and ramp-up time of 40 μm/h and 60 min, respectively.

**Figure 7:**
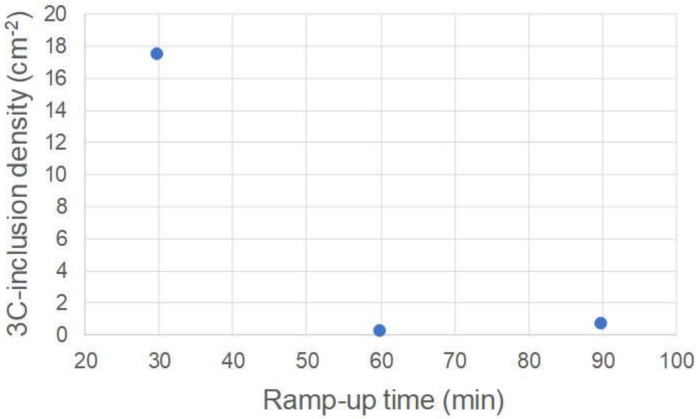
Dependence of the 3C-inclusion density of the on-axis 4H-SiC epitaxial layers at the growth rate of 40 μm/h on the ramp-up time.

**Figure 8:**
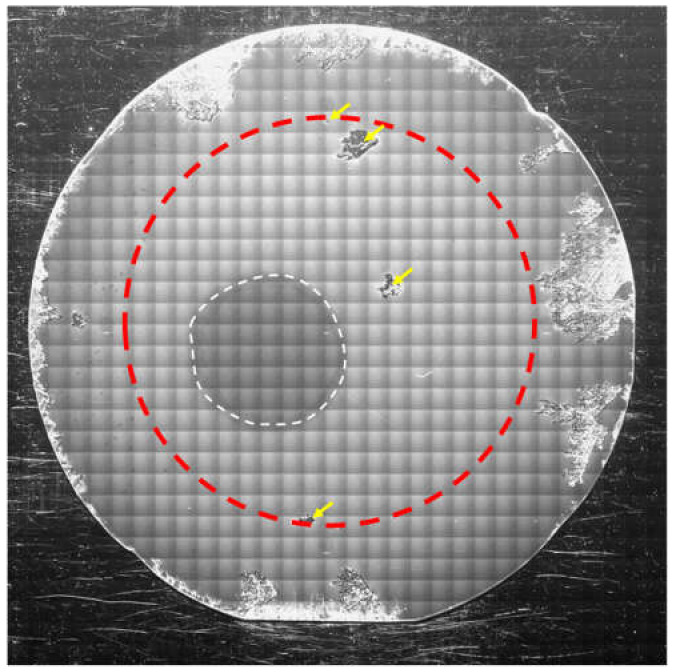
PL image of the on-axis 4H-SiC homoepitaxial layer grown on the 3-inch substrate at the growth rate and ramp-up time of 40 μm/h and 60 min, respectively.

Then, to investigate the effects of decreases in the degree of supersaturation at the initial growth stage on the 3C-inclusion density, we varied the ramp-up time between 30 and 90 min. Figure 6 shows an NOM image of the 4H-SiC epitaxial layer surface grown with a ramp-up time of 60 min. The 4H-SiC epitaxial layer surface is covered with spiral hillocks, and we confirmed that the epitaxial layers experienced spiral growth throughout the wafer irrespective of the ramp-up time. Figure 7 shows the dependence of the 3C-inclusion density on the ramp-up time. The 3C-inclusion density was calculated in concentric 2-inch areas of the 3-inch wafer, as was the case in Figure 4b. The 3C-inclusion density drastically decreases with increases in ramp-up time, and saturates when the ramp-up time is over 60 min. Figure 8 shows a PL image of the 4H-SiC epitaxial layer grown at the ramp-up time of 60 min. The 3C-inclusions are indicated using yellow arrows and the facet area of the substrate is encircled using a white dashed line. The 3C-inclusion density was 0.2 cm^−2^ at a ramp-up time of 60 min. This suggests that 3C-SiC nucleation on the terraces of the substrates was suppressed by decreasing in the degree of supersaturation at the initial growth stage. As is the case in Figure 3, high-density 3C-inclusions—which are not caused by the absence of steps at the wafer edge of the step-flow upstream side—are generated at various positions of the wafer edge. The cause of the high-density 3C-inclusions at the wafer edge is discussed in the following section.

### 3.3. Cause of the 3C-Inclusions

**Figure 9:**
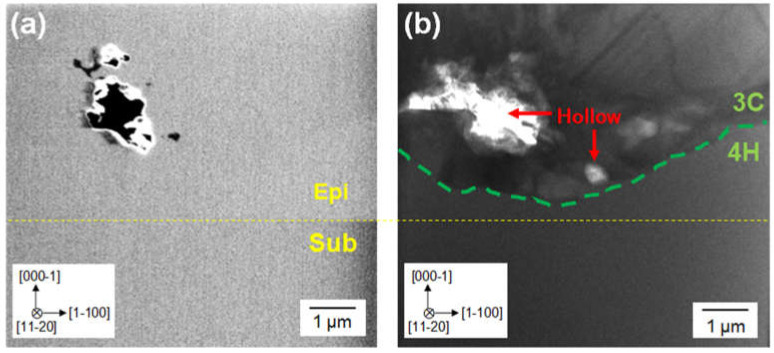
Cross-sectional (**a**) SEM and (**b**) bright-field TEM images around the origination of the 3C-inclusion in the on-axis 4H-SiC epitaxial layer at the growth rate and ramp-up time of 40 μm/h and 60 min.

**Figure 10:**
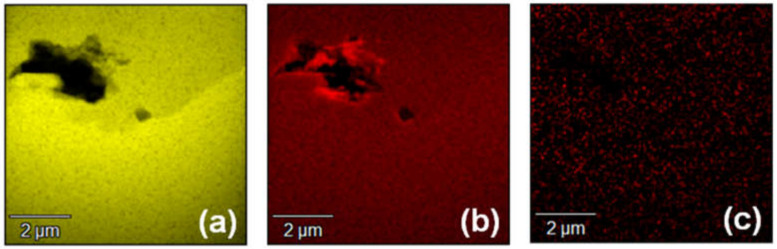
Energy-dispersive X-ray spectroscopy (EDX) maps of (**a**) Si, (**b**) C, and (**c**) Ta in the same area as that in Figure 9.

As described above, the 3C-SiC nucleation on the large terraces of the substrate surface was the cause of the 3C-inclusions. However, it is thought that other causes for the 3C-inclusions exist because the 3C-inclusion density became saturated at ramp-up times over 60 min and high-density 3C-inclusions were generated at various positions of the wafer edge. To investigate the causes of the 3C-inclusions other than the high degree of supersaturation, we observed the origination of a 3C-inclusion in an epitaxial layer grown with a ramp-up time of 60 min using cross-sectional SEM, TEM and EDX. Figure 9a,b present cross-sectional SEM and bright-field TEM images, respectively, around the origination of a 3C-inclusion. The yellow dashed lines indicate the interface between the epitaxial layer and the substrate, which was determined from the SEM contrast in Figure 9a. The interface between the 3C-inclusion and the 4H-SiC epitaxial layer is indicated by the green dashed line in Figure 9b. We found that the 3C-inclusion was generated in the epitaxial layer a few hundred nanometers from the interface between the epitaxial layer and the substrate, and hollows—indicated using red arrows—exist near the origination of the 3C-inclusion. This indicates that the cause of the 3C-inclusion was contamination by foreign materials. To predict the source of these foreign materials, we performed elemental analysis using EDX, where Si, C and Ta were detected. We assumed that contamination occurred owing to Si depositing on graphite products in the chamber, in addition to contamination due to graphite products without and with TaC-coatings—as sources for C and Ta, respectively. Figure 10a–c show EDX maps of Si, C and Ta, respectively, within the same area as that shown in Figure 9. The color intensity is strong at high-concentration areas of each element in these EDX maps. Figure 10a,c indicate that there was no high-concentration area of Si and Ta around the hollows. In contrast, Figure 10b shows that the concentration of C increased around the hollows. From these observations, it could be inferred that the graphite contamination occurred, owing to the graphite products without coatings. This contamination triggered the heterogeneous nucleation of 3C-SiC. As shown in Figure 3 and Figure 8, high-density 3C-inclusions were occasionally generated at the wafer edge. It is thought that the cause of the high-density 3C inclusions at the wafer edge was contamination owing to graphite, because—during epitaxial growth—the 4H-SiC substrates were placed on a graphite product without a coating. We inferred that the graphite contamination had a significant impact on the wafer edge. Thus, we found that the causes for the 3C-inclusions were the 3C-SiC nucleation on the large terraces of the on-axis substrates and graphite contamination. This type of contamination can be reduced by coating graphite products with SiC, TaC, or other materials to further decrease the 3C-inclusion density.

### 3.4. Growth of the 3-Inch Thick Epitaxial Layer and Free-Standing Demonstration

**Figure 11:**
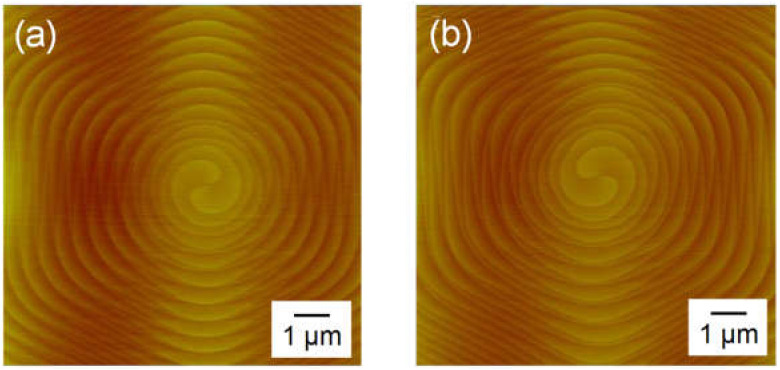
Atomic force microscopy (AFM) images of the spiral hillocks on the on-axis 4H-SiC epitaxial layers grown for (**a**) 30 min and (**b**) 5 h at the growth rate and ramp-up time of 40 μm/h and 60 min, respectively.

Applying a ramp-up time of 60 min, where 3C-SiC nucleation on the terraces of the substrates was suppressed, we grew a thick 4H-SiC epitaxial layer at a growth rate of 40 μm/h for 5 h. The thickness of the epitaxial layer was 194 μm. The 3C-inclusion density, calculated ignoring 3C-inclusions generated from the wafer edge, was 2.0 cm^−2^. This value is larger than 0.2 cm^−2^, which is the 3C-inclusion density of a thin 4H-SiC epitaxial layer at the growth time of 30 min. It is thought that the 3C-inclusion density increased with an increase in the growth time because there was constant graphite contamination during the epitaxial growth. To investigate the stability of spiral growth, we observed the morphology of spiral hillocks using AFM. Figure 11a,b show AFM images of the spiral hillocks on the 4H-SiC epitaxial layers grown for 30 min and 5 h, respectively. Both spiral hillocks have a terrace width of approximately 250 nm and a step height of 0.5 nm. The tilt angles of these spiral hillocks were calculated as approximately 0.1°. This suggests that the morphology of the spiral hillocks remained constant when the growth time increased, and stable spiral growth was achieved.

**Figure 12:**
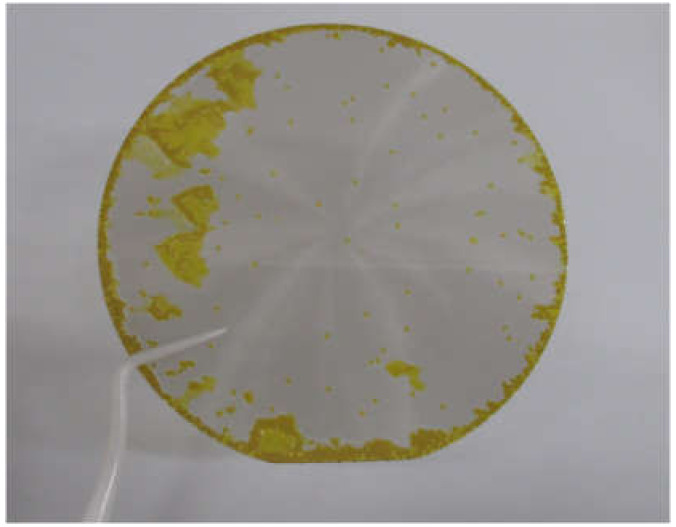
Photograph of the 3-inch on-axis 4H-SiC free-standing epitaxial layer.

Finally, we fabricated a free-standing 4H-SiC epitaxial layer. The thick 4H-SiC homoepitaxial layer grown on the 3-inch substrate with a ramp-up time of 60 min and with the thickness of 194 μm was separated from the substrate using the KABRA process. Figure 12 shows a photograph of the 3-inch free-standing 4H-SiC epitaxial layer. The epitaxial layer is transparent because of low nitrogen doping, and the yellow areas indicate 3C-inclusions. The C-face and Si-face were polished by chemical–mechanical polishing and mirror-polishing, respectively. The thickness of the free-standing 4H-SiC epitaxial layer was 182 μm, which could be handled using tweezers, as shown in Figure 12. Thus, we succeeded in fabricating a 3-inch on-axis 4H-SiC free-standing epitaxial layer with a 3C-inclusion density of 2.0 cm^−2^.

## 4. Conclusions

We investigated factors influencing the generation of the 3C-inclusions in 4H-SiC C-face on-axis epitaxial layers. We found that the addition of HCl had no effect on the 3C-inclusion density and the 3C-inclusion was suppressed by increasing the ramp-up time. The 3C-inclusion density of the 4H-SiC epitaxial layer decreased to 0.2 cm^−2^ when increasing the ramp-up time to 60 min (when the growth rate and growth time were 40 μm/h and 30 min, respectively). It is thought that the 3C-SiC nucleation on large terraces of the on-axis substrates—occurring before the substrates were covered with spiral hillocks—was suppressed. Moreover, we investigated other causes for 3C-inclusions and found that there was a high concentration of C near the origin of the 3C-inclusion. This indicates that graphite contamination from uncoated graphite products was a cause for the presence of 3C-inclusions. Finally, we succeeded in growing a thick homoepitaxial layer on the 3-inch substrate and demonstrating a free-standing epitaxial layer with a thickness of 182 μm and a 3C-inclusion density of 2.0 cm^−2^.
